# Liquid Crystal Based Binding Assay for Detecting HIV-1 Surface Glycoprotein

**DOI:** 10.3389/fchem.2021.668870

**Published:** 2021-04-26

**Authors:** Amna Didar Abbasi, Zakir Hussain, Usman Liaqat, Dooa Arif, Kun-Lin Yang

**Affiliations:** ^1^Department of Materials Engineering, School of Chemical and Materials Engineering, National University of Sciences and Technology, Islamabad, Pakistan; ^2^Department of Chemical and Biomolecular Engineering, National University of Singapore, Singapore, Singapore

**Keywords:** liquid crystals, aptamer, gp-120, binding assay, PDMS

## Abstract

Surface protein gp-120 of HIV-1 virus plays an important role in the infection of HIV-1, but detection of gp-120 during the early stage of infection is very difficult. Herein, we report a binding bioassay based on an RNA aptamer B40t77, which binds specifically to gp-120. The bioassay is built upon a hydrophobic glass slide with surface immobilized gp-120. When the glass surface is incubated in a solution containing B40t77, the aptamer is able to bind to gp-120 specifically and remove it from the surface after a short incubation time of 30 min. The result of the binding event can be amplified by using liquid crystal (LC) into optical signals in the final step. By using this bioassay, we are able to detect as low as 1 μg/ml of gp-120 with high specificity within 30 min. No response is obtained when gp-120 is replaced by other protein such as bovine serum albumin (BSA). This is the first qualitative bioassay which provides a simple way for the detection of gp-120 with the naked eye. The assay is robust, low-cost and does not require additional labeling. Thus, the bioassay is potentially useful for the early detection of HIV-1 in resources-limited regions.

## Introduction

Gp-120 is a surface glycoprotein of HIV-1 virion. It plays a central role in early immune dysfunction which is a characteristic of HIV-1 infection (Rychert et al., 2010). It is able to recognize and interact with specific receptors (e.g., CD4 in combination with either CCR5 or CXCR4) present on target cells (Wyatt and Sodroski, 1998; Banerjee and Mukhopadhyay, 2016). Therefore, targeting this step of gp-120 and CD4 interaction is very critical for diagnosis, inhibition and therapeutics of HIV-1 (Galdiero et al., 2011). Even though antibody-based immunoassays have been widely used in the detection of HIV-1, they do not allow early detection of HIV-1 due to low levels of antibodies present in the early stage of HIV-1 infection (Jangam et al., 2013). Detection HIV-1 infection in the so-called “window period” requires an analytical method that does not rely on antibodies (Branson and Stekler, 2012). Although there are molecular techniques that are readily available for the detection of gp-120, such as enzyme linked immunoassay (ELISA) (Rychert et al., 2010) and western blotting (Lee et al., 2000). However, these methods are very time-consuming, costly and require additional labeling, experienced personnel and well-equipped laboratories (Tsang et al., 2018). Therefore, there is a need to develop a new analytical method which is sensitive, simple, label-free and economical for the detection of gp-120.

Recently, aptamers have been exploited as molecular probes in various types of bioassays due to their high specificity, low cost, thermal stability, and ease of modification (Huang et al., 2015; Wen et al., 2015; Rahimizadeh et al., 2017). Aptamers are synthetic single-strand oligonucleotides (DNA or RNA) which bind to specific target molecules with high affinity (Robertson and Joyce, 1990; Tuerk and Gold, 1990). Because of their properties, aptamers can replace antibodies in various bioassays. For the diagnosis of HIV-1, aptamers are able to detect both early (viral genes and gp-120 protein) and late (antibodies) infection markers (Wandtke et al., 2015). In the literature, Dey et al. in 2005 isolated and characterized two anti-gp-120 RNA aptamers B40 and B40t77. Both of them are able to neutralize R5 strain of HIV-1 by binding specifically to its surface gp-120 (Dey et al., 2005). Recently, John et al. in 2014 presented an electrochemical biosensor based on 4 poly(propylene imine) dendrimer/streptavidin platform for immobilization of B40 aptamer to detect gp-120 (John et al., 2014). However, B40 is susceptible to nucleases and that caused some reliability issues. In contrast, B40t77 (77 nucleotide-long, the truncated form of B40) is resistant to nuclease because its 2' position of nucleobases is substituted by fluoro, amino or O-methyl groups (Osborne and Ellington, 1997; Dey et al., 2005). Despite of its short length, it is able to neutralize infectivity of HIV-1 R5 strains by binding specifically to its surface gp-120 and blocking viral entry (Dey et al., 2005). However, B40t77 has not been used for the development of a bioassay for gp-120 so far, because it was created for the purpose of HIV-1 therapeutics initially. In this study, we chose B40t77 as a molecular probe to develop a bioassay for detecting gp-120.

In traditional bioassays, detection signal was amplified by using fluorescence or enzyme-linked antibodies. However, these approaches require labeling of biomolecules with additional probes (fluorophores or enzymes). Recently, liquid crystals received much attention due to their unique optical properties and long-range orientational orders (Li et al., 2014; Hussain et al., 2016; Kim et al., 2018). Many research groups have exploited orientational response of liquid crystal to surface-bound biomolecules as a mechanism to develop various bioassays and biosensors which enable naked eye detection. In the literature, it has been reported that the orientational response of liquid crystal can be amplified by an intrinsic cooperative behavior associated with long-range interactions. The orientational response of liquid crystal also produces optical readouts which are visible to the naked eye under crossed polarizers. Thus, LC-based biosensors eliminate the need of labeling, amplification, and expensive instrumentation (Hussain et al., 2016). It can be used as a portable biosensor in resource-limited regions. Previously, there are many reports of LC-based biosensors for the detection of bacteria (Xu et al., 2010; Zafiu et al., 2016), viruses (Han et al., 2014), enzymes (Hu and Jang, 2012), proteins (Xue and Yang, 2008; Alino et al., 2012; Kim et al., 2018; Kim and Jang, 2019), organophosphate (Yang, 2005), DNA (Yang, 2005; Price and Schwartz, 2008; Chen and Yang, 2010), and glucose (Zhong and Jang, 2014) etc., but it has not been used for the detection of gp-120 due to the lack of specific molecular probes.

In this paper, we aim to combine liquid crystal and the molecular probe B40t77 to develop a bioassay which is suitable for the detection of gp-120. The bioassay consists of a hydrophobic glass slide, which allows non-specific adsorption of gp-120 on the surface. Liquid crystal is applied to the surface to report the spatial distribution of gp-120 on the surface. In an event that the glass surface is incubated in a solution containing B40t77 aptamer, we hypothesize that the aptamer is able to bind to gp-120 and remove it from the surface. The binding event can be probed by using LC after the incubation step. Recently, integration of biosensing platforms into lab-on-a-chip systems by introducing microfluidics has been an increasing interest (Haeberle and Zengerle, 2007). As previously reported, microfluidic based bioassays have attracted a lot of attention due to their high sensitivity, fast response and short assay time due to their large surface-to-volume ratios (Sia and Whitesides, 2003). In this study, we also aimed to combine the bioassay with a microfluidic device to shorten the response time of the bioassay. This is the first report showing the application of LC and aptamers combined together for the detection of gp-120 and potentially for the early detection of HIV-1.

## Experimental Section

### Materials

*N, N*-dimethyl-*n*-octadecyl-3aminopropyltrimethoxy-silyl chloride (DMOAP), bovine serum albumin (BSA), RNase free water, RNase ZAP and magnesium chloride were purchased from Sigma Aldrich (Singapore) and used as received. Microscopic glass slides were obtained from Merienfield (Germany). Liquid crystal 4-cyano-4-pentylbiphenyl (5CB) was purchased from Merck (Japan). Tris EDTA (1 × TE, pH 8) and PBS buffer (10 ×, pH 7.4) were purchased from 1st BASE (Singapore). TEM grids (copper, 100 parallel lines) were obtained from Electron Microscopy Sciences (U.S.A). Sylgard 184 (PDMS monomer and curing agent) was purchased from Dow Corning (U.S.A). Decon-90 was purchased from VWR (Singapore). Mylar transparency mask was purchased from Infinite Graphics (Singapore). Silicon tubing (ID 0.031× OD 0.094 inches) was purchased from Cole Parmer (U.S.A). Protein gp-120 was purchased from Abcam (U.K.). 2-fluoro pyrimidine substituted 77 nucleotides long B40t77 RNA aptamer was custom synthesized by Gene Link (U.S.A). Apt 8 (anti-IgG Aptamer) was purchased from IDT (Singapore). Aptamer stock solution was prepared by dissolving B40t77 in RNase-free water at room temperature and diluted by using TE buffer with 100 mM of magnesium chloride. Prior to use, the aptamer solution was heated to 95°C for 3 min, cooled at room temperature for 5 min and then cooled on ice to ensure correct spatial folding of the aptamer. All buffer solutions were prepared in 0.2 μm filtered milli Q water.

### Chemical Modification of Glass Slides

Glass slides were first rinsed with deionized water two times and then immersed in a 5% (v/v) Decon-90 solution overnight. Then, the glass slides were cleaned ultrasonically in deionized water for 15 min and rinsed with copious amount of deionized water. To prepare DMOAP-coated glass slides, the cleaned glass slides were immersed in a 0.1% (v/v) DMOAP solution for 5 min. Subsequently, the slides were rinsed with deionized water five times to remove unreacted DMOAP from the surface and then dried under a stream of compressed nitrogen gas. Finally, the immobilized DMOAP was cross-linked in a vacuum oven at 100°C for 15 min.

### Immobilization of gp-120 on a DMOAP-Coated Slide

**Figure 1:**
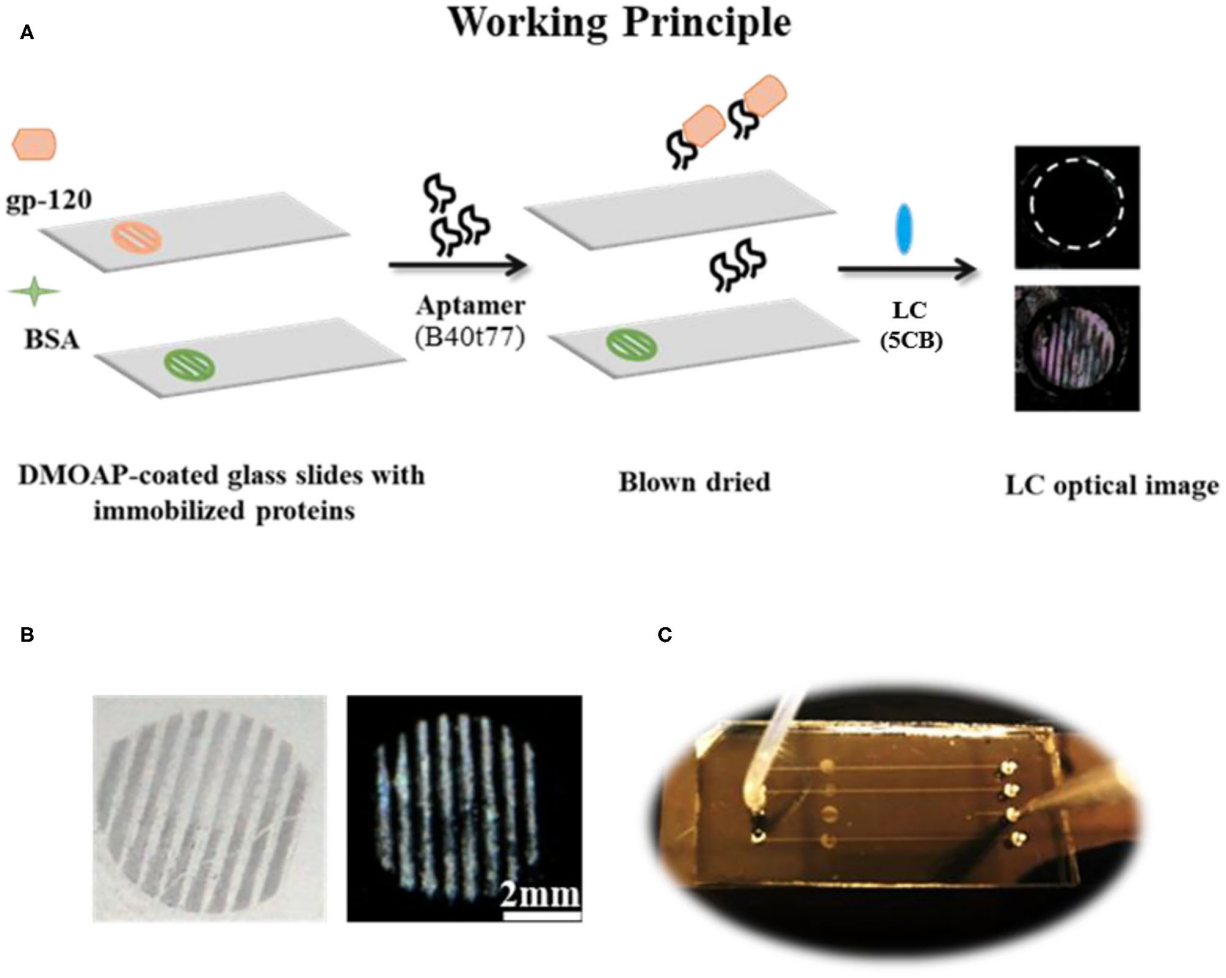
**(A)** The working principle for the detection of gp-120 by using an RNA aptamer B40t77 and LC. **(B)** Optical images of surface-immobilized gp-120 (left) and corresponding LC images (right) showing that the pattern of surface-immobilized gp-120 on the glass slide closely ensembles a TEM grid. **(C)** Top view of a microfluidic device for the proposed binding assay. The inlet of the microfluidic device was connected to a piece of silicon tubing for delivering a solution containing B40t77 at a controlled flow rate.

All stock and working solutions of gp-120 and BSA were prepared in 0.01 M of phosphate buffered saline (PBS, pH 7.4). Next, TEM copper grids with parallel lines were used as a template to generate parallel protein lines on DMOAP coated glass slides as shown in Figure 1B. The TEM copper grids were cleaned by sonication with ethanol, methanol and acetone for 15 min sequentially followed by drying in an oven at 100°C overnight. To immobilize gp-120, a TEM copper grid was placed on a DMOAP-coated slide, and gp-120 solution with a concentration of 1 μg/ml was pipetted to cover the entire grid (~2 μl). After 1 h of incubation in a humidified chamber, the glass slide was blown dried under a stream of nitrogen and the TEM grid was removed with the help of a micropipette. To check the specificity and cross reactivity of proposed bioassay, patterns of BSA were also prepared by following the same procedure as described for gp-120, except that the BSA concentration was 6 μg/ml.

### Fabrication of Microfluidic Devices

The surface of PDMS with embossed microfluidic channels was prepared by casting Sylgard 184 on a silicon master with raised features (Width × Depth × Length/0.2 × 0.04 × 40 mm). Silicon master was fabricated by defining the channel patterns through photolithography onto a negative photoresist (SU-8). Microfluidic channels were then molded from PDMS using standard soft lithography techniques (Xue et al., 2009; Alino et al., 2012). In brief, silicon elastomer and curing agent were mixed in a weight ratio of 10:1 and the mixture was degassed for 60 min after pouring on silicon master to remove air bubbles. After degassing, PDMS was cured at 65°C for 4 h. Finally, the cured PDMS with micro-channels was carefully peeled off from silicon master and cut into appropriate size. Holes were punched out on the PDMS to form inlets and outlets. To clean the PDMS, it was soaked in absolute ethanol overnight, washed with deionized water and dried under a stream of nitrogen. Finally, a closed microfluidic device was prepared by binding the PDMS with a piece of DMOAP-coated slide with surface-immobilized gp-120. The microfluidic channel was carefully aligned with the immobilized gp-120 on the surface as shown in Figure 1C. To ensure good sealing, pressure was applied to the system for 30 s.

### Removal of Surface-Immobilized gp-120

The gp-120 adsorbed glass slides in parallel patterns was incubated with different concentrations (10, 16, 20, and 40 μg/ml) of B40t77 (~2 μl) aqueous solution for 1 h at room temperature in a home-made humidified chamber to allow B40t77 to bind with adsorbed gp-120 on glass slide and subsequently pulling these proteins off the surface of glass slide. Thereafter, these slides were rinsed with filtered Milli Q water and dried under a stream of nitrogen gas. Finally, results were examined by using LC as signal reporter as depicted in working principle shown in Figure 1A.

### LC-Based Binding Bioassay by Using Microfluidic Device and Syringe Pump

To further streamline the assay, we used microfluidic flow channel device which is connected to the syringe pump (PHD Ultra, Harvard) through silicone tubing. Fifteen microliter of aptamer solution with different concentrations was flowed through the micro-channels by using the infuse function of the syringe pump. The volume of the aptamer solution was controlled by using the withdrawal function of syringe pump. After washing the micro-channels with 2 μl of water under a continuous flow mode, the PDMS microfluidic device was peeled off from glass slide followed by blow drying of the sample glass slide under stream of compressed nitrogen gas. Finally, results of this assay were analyzed by using LC as signal reporter.

### Preparation of LC Optical Cells for Imaging Protein Patterns

A piece of sample glass slide and a piece of DMOAP-coated slide were used to prepare an LC optical cell. The two glass slides were put together and separated by Mylar films (thickness ~6 μm) at both ends of the glass slides. The optical cell was secured at both ends with binder clips. Approximately 10 μl of LC was loaded into the empty space between the two glass slides by using capillary force. After 15 min, the prepared LC optical cell was analyzed by using 1x objective lens under a polarized optical microscope Nikon ECLIPSE LV100POL (Japan) in the transmission mode.

## Results and Discussion

### Detection of Surface-Immobilized gp-120 Using LC

**Figure 2:**
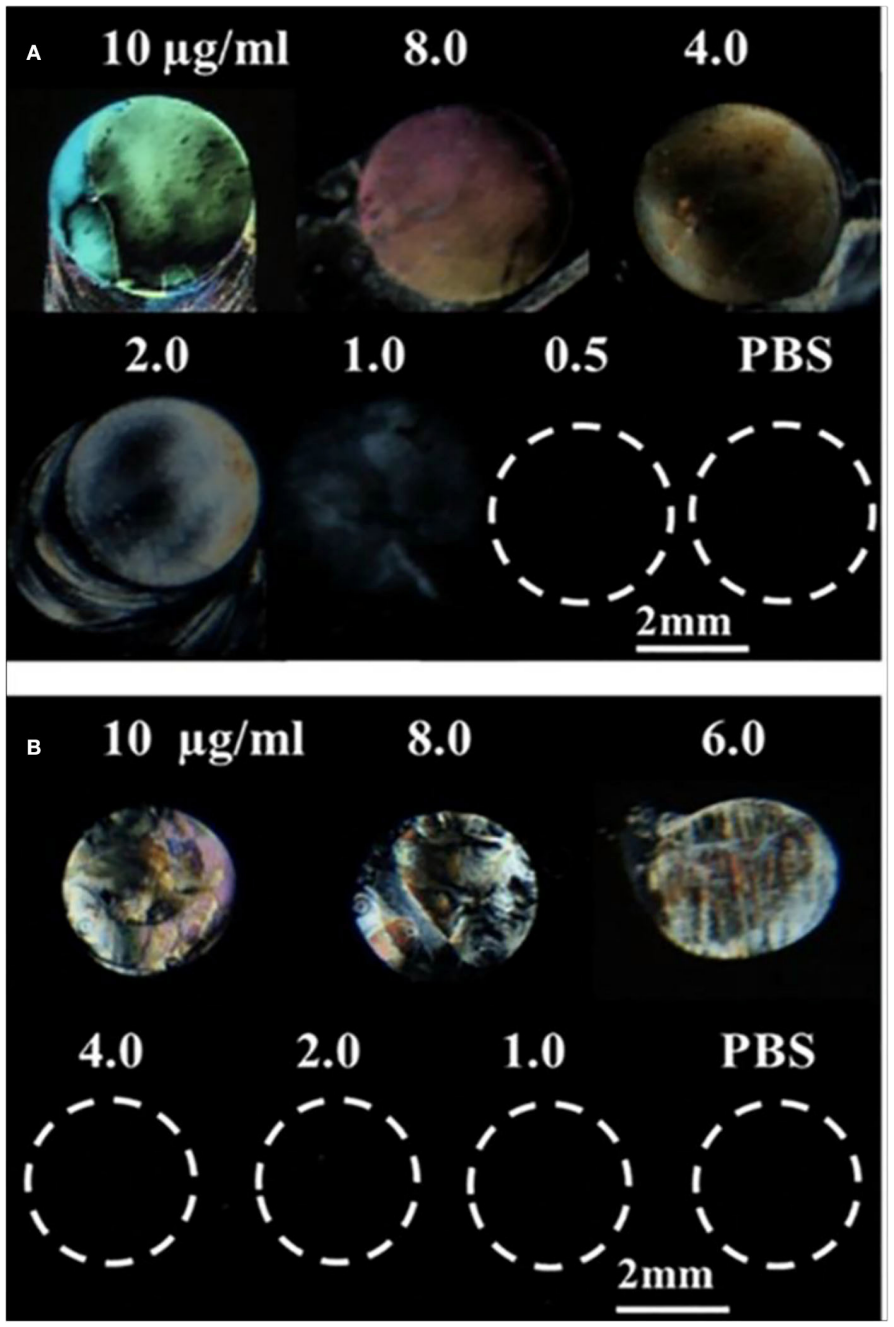
Optical images of LCs supported on a DMOAP-coated surface immobilized with different concentrations of **(A)** gp-120 and **(B)** BSA. Number above each spot is the protein concentration (μg/ml).

We first investigated whether LC can be used to detect surface-immobilized gp-120 and effect of gp-120 concentrations on the LC optical responses. In this experiment, a piece of DMOAP-coated glass slide was chosen as a platform to immobilize gp-120. In literatures, the DMOAP-coated slide has been widely used to control the orientations of LC and for immobilization of biomolecules (Naemura, 1980; Xue and Yang, 2008). To understand the effect of gp-120 on the orientations of LC, we spotted droplets of gp-120 solution (~0.5 μl) with different concentrations on the DMOAP-coated slide in an array format. After 1 h of incubation in a humidified chamber at room temperature, the slide was blown-dried and used to make an LC optical cell. Optical images of LC under crossed polarizers were shown in Figure 2A. It can be seen that the orientation of LC was disrupted by the surface immobilized gp-120 and gave a bright spot only when the concentration of gp-120 was 1 μg/ml or higher. In other regions without any gp-120 or in regions where the gp-120 concentration was lower than 1 μg/ml, the orientation of LC was not disrupted, as is evident by the dark color. This result suggests that the lowest concentration of gp-120 which can disrupt the orientation of LC on the DMOAP-coated slide is 1 μg/ml. Moreover, Figure 2A shows that the degree of disruption and brightness increased with increasing concentration of gp-120 from 1 to 10 μg/ml. In the literature, the interference color of LC can be used as a means to report concentration of an analyte (Chen and Yang, 2012). Herein, we also observed that a higher concentration (10–2 μg/ml) led to a bright color whereas a low concentration (1 μg/ml) led to a white or gray color. For comparison, we also spotted different concentrations of BSA and observed how they have disrupted the orientations of LC. Figure 2B shows that the lowest concentration of BSA which can disrupt LC and gave a bright signal is 6 μg/ml. When the concentration of BSA was lower than 6 μg/ml, the LC remained dark, because the surface density of BSA was too low to disrupt LC orientation. The lowest concentrations (gp-120, 1 μg/ml and BSA, 6 μg/ml) which can disrupt LC were chosen to generate parallel lines pattern of gp-120 and BSA for subsequent experiments of competitive binding bioassay.

### Removal of Surface-Immobilized gp-120 by Using B40t77

**Figure 3:**
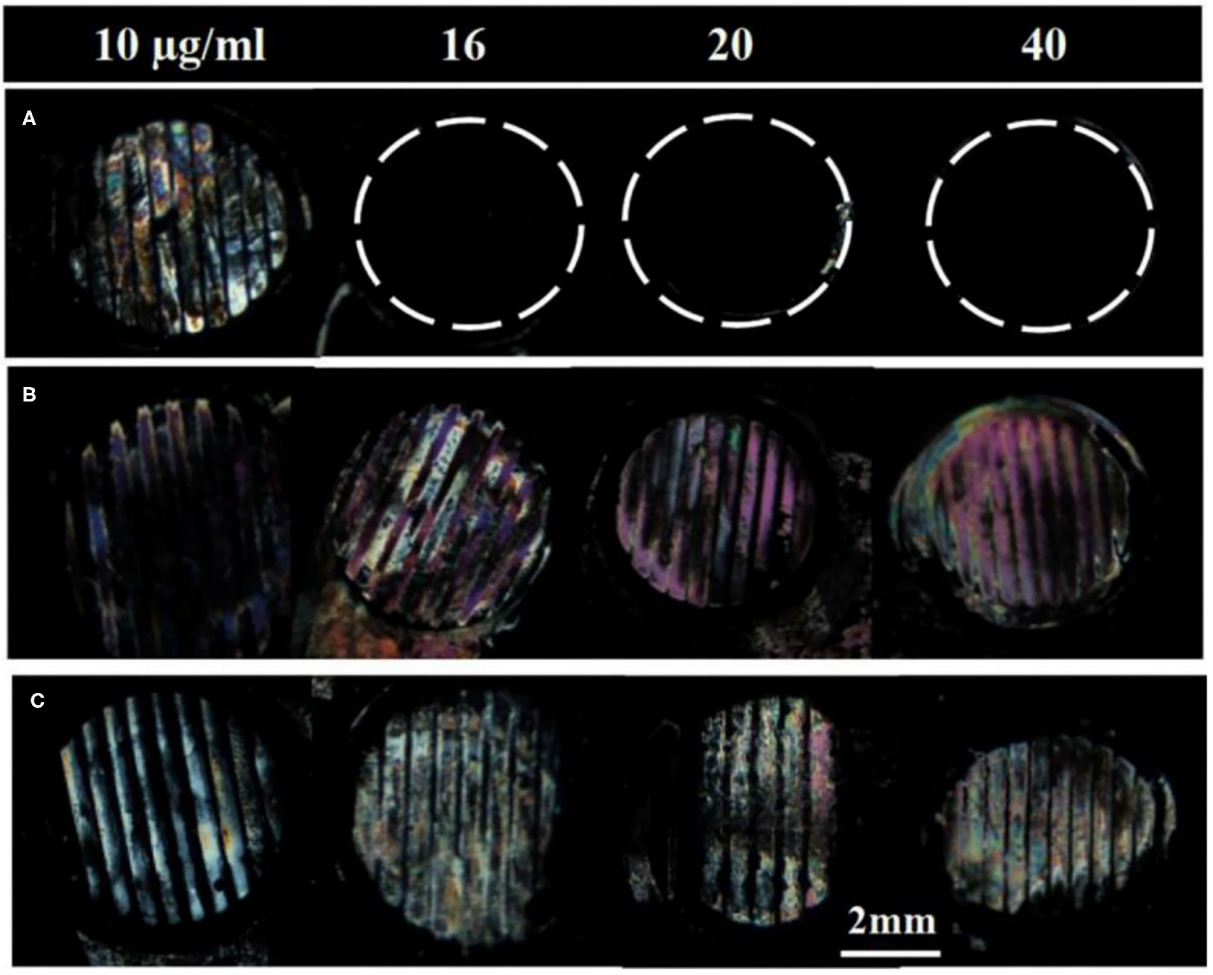
Effect of aptamers solution concentrations on the removal of surface-immobilized **(A)** gp-120, **(B)** BSA, and **(C)** gp-120. Numbers above are concentration of Aptamer solution used in the study, B40t77 in **(A,B)** whereas Apt 8 in case of **(C)**. Optical images of LC show that surface-immobilized gp-120 can be released from the surface only when the concentration of B40t77 was 16 μg/ml or higher whereas bright images were observed in case of BSA treated with B40t77and gp-120 treated with Apt 8 instead of B40t77.

Next, we investigated if the surface-immobilized gp-120 can be removed by incubating it in a B40t77 solution. Firstly, a TEM grid was used as a template to immobilize gp-120 on a DMOAP-coated glass slide. Successful immobilization of gp-120 was confirmed by using the optical image of LC as shown in Figure 1B. The line pattern of the surface-immobilized gp-120 resembled the shape of the TEM grid. Following the procedure, ~2 μl of a solution containing B40t77 was dispensed onto the surface to cover the immobilized gp-120 completely. After 1 h of incubation, the solution was blown dried and LC was used to examine the remaining gp-120 pattern on the surface. Figure 3A shows that when the B40t77 concentration was 40 μg/ml, no gp-120 could be detected. This result suggests that B40t77 is able to bind to the surface-immobilized gp-120 and remove it from the surface. The result was expected because the B40t77 was specifically designed and constructed for binding gp-120 with high affinity (Dey et al., 2005). From Figure 3A, it can also be seen that the binding event is concentration-dependent. When the concentration of B40t77 was decreased to 16 μg/ml, no gp-120 could be detected. At this condition, the amount of B40t77 was still sufficient to reduce the density of surface-bound gp-120 below the limit of detection. However, when the B40t77 concentration was further reduced to 10 μg/ml, LC gave a bright signal which can be easily distinguished from the dark background. The bright signal was caused by gp-120 which remained on the surface. Thus, it was proposed that 16 μg/ml is the minimal B40t77 concentration which is needed to clear surface-immobilized gp-120. Whereas, in Figure 3B, B40t77 shows no binding for BSA, even if the B40t77 concentration was 40 μg/ml. As a result, remaining BSA on the surface was still able to disrupt the orientation of LC supported on DMOAP-coated slide and gave a bright spot. To test binding specificity, we replaced the B40t77 solution with an anti-IgG aptamer (Apt 8) solution at four different concentrations. As shown in Figure 3C, no interaction of Apt 8 with gp-120 was found in these control experiments. LC was still disrupted by surface-bound gp-120 after the incubation.

**Figure 4:**
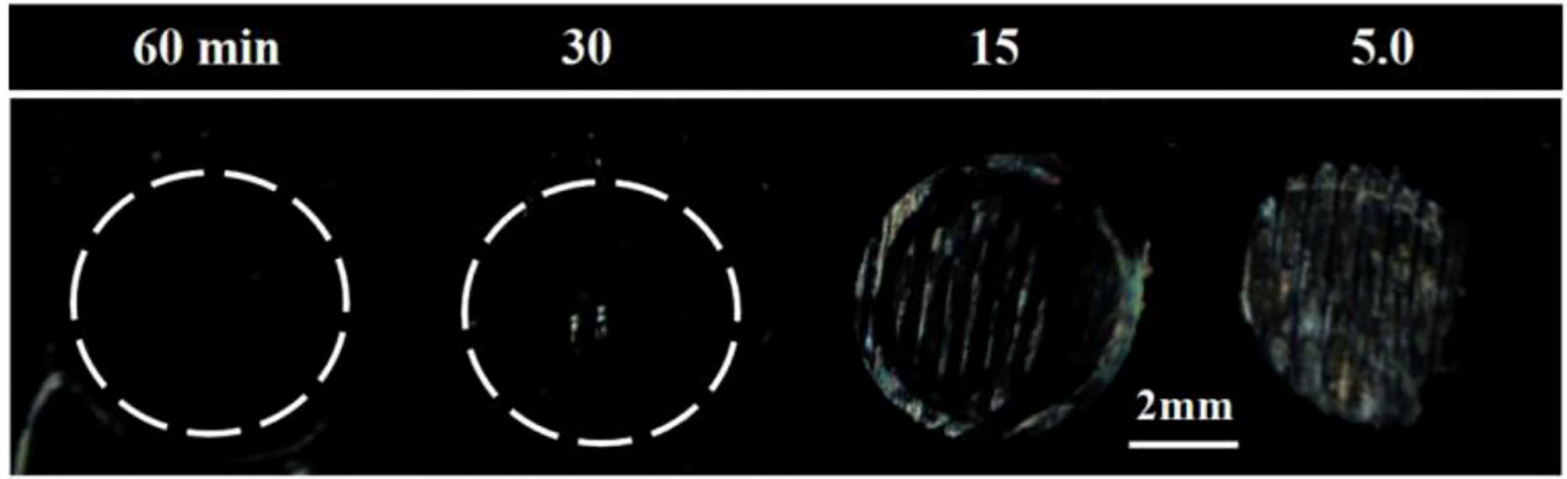
Effect of incubation time on removal of surface-immobilized gp-120. Optical images of LC supported on DMOAP coated glass slide decorated with gp-120 patterns after incubation in a B40t77 solution (16 μg/ml) for different incubation times (60, 30, 15, and 5 min).

To investigate whether the incubation time also played a role in the binding event, we varied the incubation time from 5 to 60 min while fixing the B40t77 concentration at 16 μg/ml. As shown in Figure 4, an incubation time of 60 min was required; otherwise, the surface-immobilized gp-120 could not be removed completely by using B40t77. A shorter incubation time led to incomplete removal of surface-immobilized gp-120, as shown in Figure 4.

### Development of a Microfluidic Bioassay

**Figure 5:**
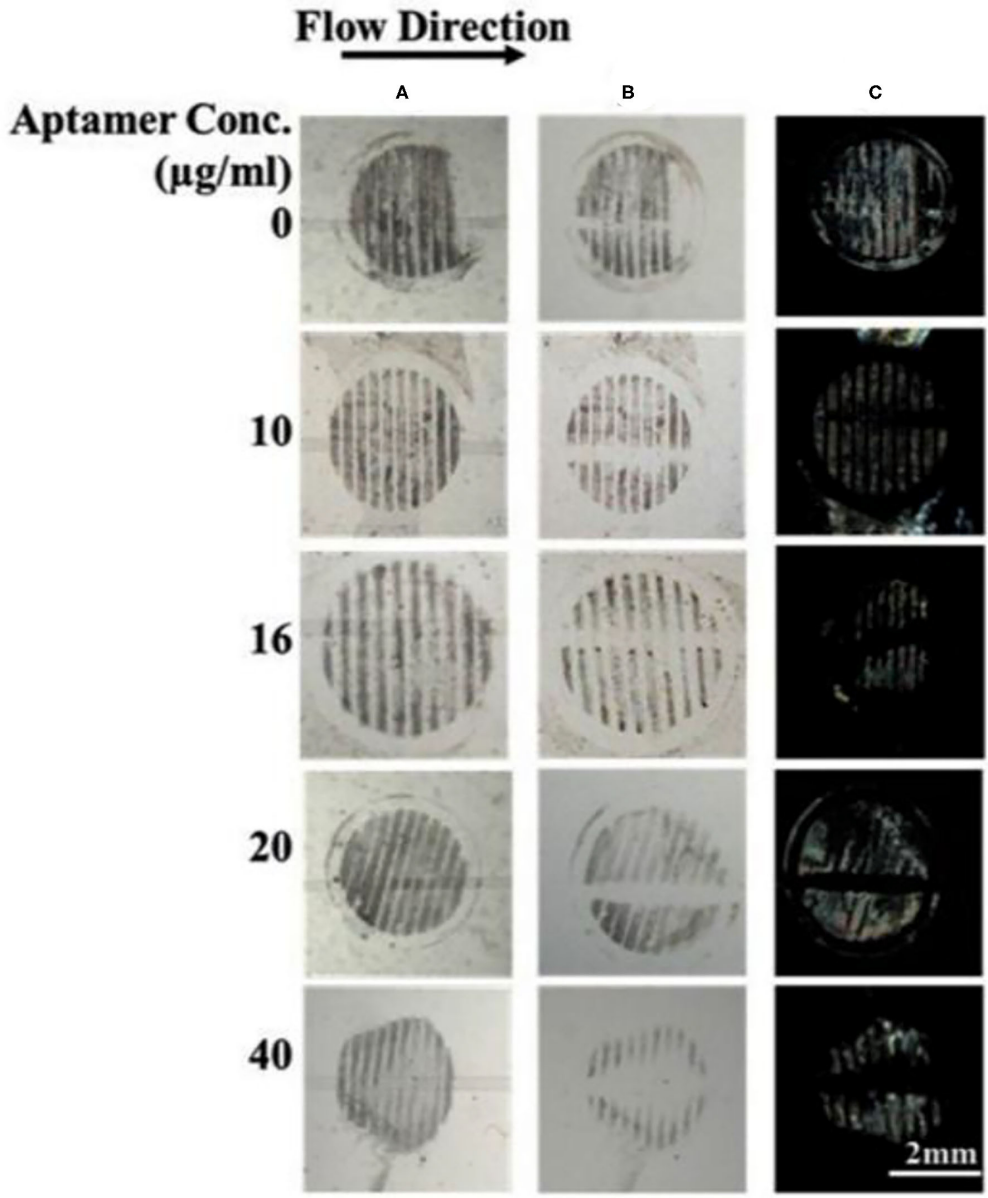
Effect of aptamer concentration on the removal of surface-immobilized gp-120 from a DMOAP-coated surface. **(A,B)** Optical images of gp-120 patterns before and after flowing of different concentrations of B40t77 (0, 10, 16, 20, and 40 μg/ml), respectively. **(C)** Optical images of LC after the assay was completed. The flow rate was 0.5 μl/min.

To streamline the procedures, we further developed a binding assay for the detection of gp-120 in a microfluidic device. Firstly, gp-120 was patterned on the surface as shown in Figure 5A. Next, we prepared a closed microfluidic channel by aligning an open PDMS micro-channel with the surface-immobilized gp-120 pattern on a DMOAP-coated slide by physical attachment as shown in Figure 1C, Fifteen microliters of TE buffer containing aptamer with different concentrations (0, 10, 16, 20, and 40 μg/ml) were flowed at a constant flow rate of 0.5 μl/min through the micro-channel. The total assay time was 30 min. As shown in Figure 5B, surface-patterned gp-120 can be removed after the aptamer solution passed through the micro-channel. The same result can be obtained by using LC as a signal reporter as shown in Figure 5C. From the results, it can be concluded that aptamer concentration at 16 μg/ml or higher could lead to complete removal of surface-immobilized gp-120 from the flow path. However, when the aptamer concentration was 10 μg/ml, the surface-immobilized gp-120 could be removed only partially. Moreover, in the case of TE buffer with no aptamer, we found that the pattern of gp-120 before and after the flow remained unchanged. These results clearly showed that the removal of surface-immobilized gp-120 (1 μg/ml) was caused by binding of B40t77 and gp-120. Furthermore, the comparison of the present work with already available HIV-1 gp-120 detection methods in literature is listed in Table 1. Despite of the fact that the limit of detection (LoD) is not the lowest but the present biosensing technique is easy to operate, simple and requires minimal sample volume as compared to other methods e.g., ELISA.

**Table 1:** Comparison of present work with available HIV-1 detection techniques.

**Method**	**Matrix**	**LoD (ng/ml)**	**References**
ELISA	Blood	0.5	Rychert et al., 2010
Plasmonic ELISA	Physiological buffer	10^−8^	Cecchin et al., 2014
Microcantilever deflection	Physiological buffer	8× 10^3^	Lam et al., 2006
Laser-induced fluorescence detection	Physiological buffer	120	Zhou et al., 2000
LC- based binding assay	Physiological buffer	1× 10^3^	This work

### Effect of Flow Rate

**Figure 6:**
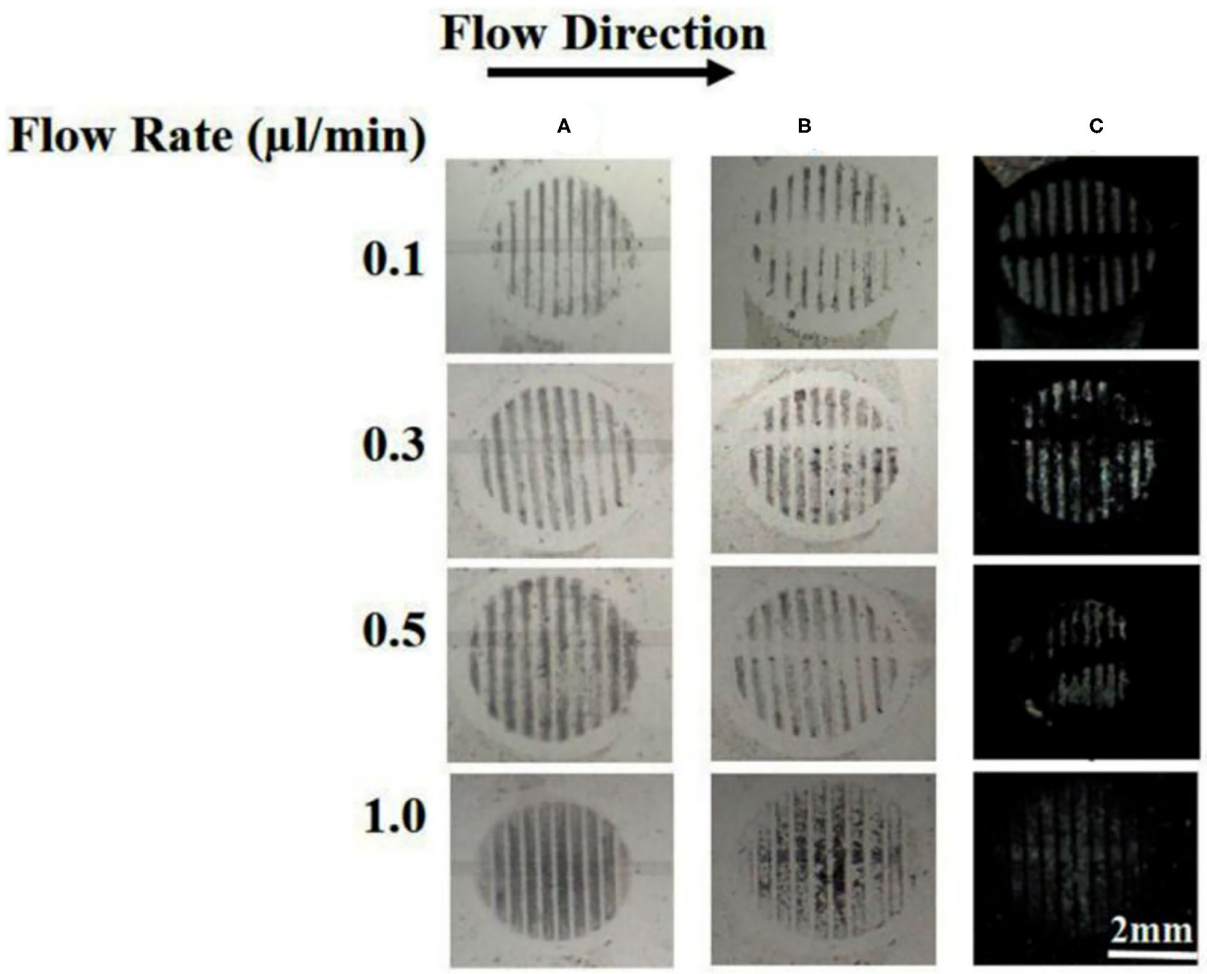
Effect of flow rate on the removal of surface-immobilized gp-120 from a DMOAP-coated surface by using B40t77. **(A)** Before flowing B4077 solution. **(B)** After flowing B40t77 solution at different flow rates (0.1, 0.3, 0.5, and 1 μl/min). **(C)** Optical images of LC after the assay was completed. The B40t77 concentration used was 16 μl/ml and the sample volume was 15 μl.

One of the advantages of using a microfluidic device to deliver B40t77 is that the flow rate can be controlled precisely by using a syringe pump. To check the effect of flow rate on the performance of the assay, we repeated the experiment by varying the flow rates from 0.1 to 1 μl/min. The total B40t77 solution volume was fixed at 15 μl and concentration used was 16 μg/ml. From Table 2, it is apparent that a higher flow rate leads to a decrease in the residence time (Aubin et al., 2009). As shown in Figure 6, when the flow rate was in the range of 0.1–0.5 μl/min, the surface-immobilized gp-120 was completely cleared by the flowing solution inside the flow path due to the interaction of B40t77 with target protein gp-120. On the other hand, flow rate was >0.5 μl/min leads to assay failure as shown in Figure 6, when flow rate was 1.0 μl/min; the surface-immobilized gp-120 remained on the surface. On the basis of our observation during the experiment, a higher flow rate inevitably led to solution leakage from the side of the micro-channel. Thus, the surface-immobilized gp-120 could not be removed by the aptamer solution. These results suggest that a low flow rate is better than high flow rate. However, a very low flow rate at 0.1 μl/min led to a long assay time which is also not desirable. These results, when combined, suggest that a flow rate of 0.5 μl/min is suitable for the assay.

**Table 2:** Residence time, time to complete assay, and working outcomes of bioassay under different flow rates.

**Volumetric flow rate (μl/min)**	**Residence time (sec)**	**Total assay time (min)**	**Assay works or not (Yes/No)**
0.1	180	150	Yes
0.3	60	50	Yes
0.5	36	30	Yes
1	18	15	No

### Specificity, Stability, and Reproducibility

**Figure 7:**
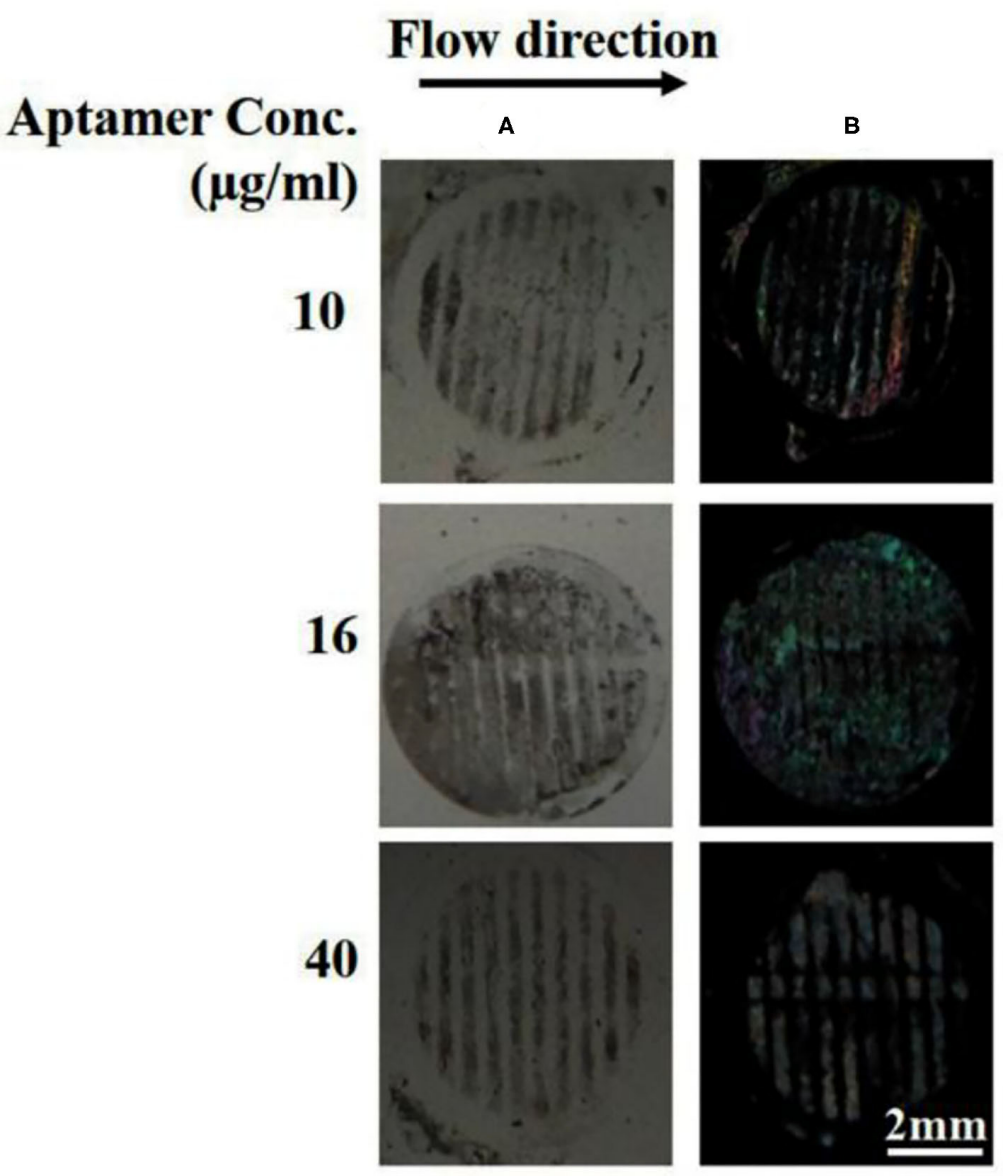
Specificity by using BSA protein as a control experiment. **(A)** Optical image of BSA patterns after flowing different concentrations of B40t77 (10, 16, and 40 μg/ml). **(B)** Optical images of LC after the assay was completed. The flow rate was 0.5 μl/min.

One of the reasons to choose B40t77 as a molecular probe for detecting gp-120 protein was the high specificity of B40t77 toward gp-120. To test the specificity, we used BSA to check cross reactivity of the proposed bioassay. All experimental conditions and procedures were same except that gp-120 was replaced by BSA. Next, solutions containing different concentrations of aptamer (10, 16, and 40 μg/ml) were flowed through the surface coated with BSA. Figure 7 shows that the surface pattern of BSA before and after the flow of B40t77 were same, indicating that the aptamer did not interact with BSA even at the highest concentration (40 μg/ml) tested. As a result, it can be concluded that the bioassay shows no cross-reactivity against other proteins such as BSA.

Due to various influencing factors, the stability and reproducibility of biosensors are worthy of research attention. In order to make our microfluidic device more stable and reproducible all the influencing factors were kept same while preparing sensor substrate. We observed that the protein pattern on our substrate remained stable for more than 2 weeks at 4°C and can be used to prepare closed microfluidic flow channel device by placing PDMS flow channels on dried glass substrate with little pressure for 30 s. Moreover, the thickness of LC film is also of keen interest, we have used mylar film (~6 μm) while making the LC optical cell. All experiments were carried out at room temperature and the flow rate and reaction time were also kept constant after optimization.

## Conclusions

In summary, we have successfully developed a binding bioassay for gp-120 by using aptamer B40t77 as a molecular probe and liquid crystal as a signal reporter. This bioassay shows high specificity against gp-120 without any cross-reactivity with BSA. The bioassay was integrated with a microfluidic device to control the flow rate and the total assay time was 30 min. At the optimal flow rate of 0.5 μl/min, the surface-immobilized gp-120 can be released from the surface by flowing 16 μg/ml of B40t77 through binding events. For detection purposes, this event can be transduced and amplified by using liquid crystal into optical signal visible with the naked eye. The simple and low-cost microfluidic device has the potential to detect HIV-1 in early stages.

## Data Availability Statement

The original contributions presented in the study are included in the article/supplementary material, further inquiries can be directed to the corresponding author.

## Author Contributions

AA: experimentation, data acquisition, and write up. DA: data acquisition and manuscript preparation. UL: data interpretation and manuscript corrections. K-LY: supervision, proof reading, and data interpretation. ZH: conceptualization, supervision, data interpretation, and proof reading. All authors contributed to the article and approved the submitted version.

## Conflict of Interest

The authors declare that the research was conducted in the absence of any commercial or financial relationships that could be construed as a potential conflict of interest.
